# A Qualitative In Vitro SEM Study on the Protective Effects of a Self-Antibacterial Nano-Hydroxyapatite Toothpaste Against Acid-Induced Enamel Surface Erosion

**DOI:** 10.3390/ijms27062796

**Published:** 2026-03-19

**Authors:** Chamnan Randorn, Pongpen Kaewdee, Gobwute Rujijanagul, Sujitra Tandorn

**Affiliations:** 1 Department of Chemistry, Faculty of Science, Chiang Mai University, Chiang Mai 50200, Thailand; chamnan.r@cmu.ac.th (C.R.); pongpen.k@cmu.ac.th (P.K.); 2 Center of Excellence in Materials Science and Technology, Chiang Mai University, Chiang Mai 50200, Thailand; gobwute.ruji@cmu.ac.th; 3 Office of Research Administration, Chiang Mai University, Chiang Mai 50200, Thailand; 4 Department of Physics and Materials Science, Faculty of Science, Chiang Mai University, Chiang Mai 50200, Thailand; 5 Multidisciplinary Research Institute, Chiang Mai University, Chiang Mai 50200, Thailand

**Keywords:** antibacterial activity, demineralization, enamel, nano-hydroxyapatite, remineralization

## Abstract

This study evaluated the potential protective effect of a synthesized self-antibacterial nano-hydroxyapatite (nano-HA) toothpaste against erosive changes in the enamel surface induced by a cola-based soft drink, based on a qualitative scanning electron microscopy (SEM) study, in comparison with conventional fluoride toothpaste. Thirty extracted human premolars were sectioned to obtain enamel specimens and randomly assigned into a control group and experimental groups in which fluoride or synthesized nano-HA toothpaste was applied either before or after cola exposure (*n* = 5 per group). Enamel surface morphology was qualitatively assessed using SEM, and surface roughness (Ra) was estimated using a semi-quantitative approach based on SEM image analysis using ImageJ software. Antibacterial activity was evaluated using the agar diffusion method to explore the potential additional benefits of the synthesized self-antibacterial nano-HA formulation. SEM observations showed that, after cola exposure, specimens treated with nano-HA exhibited less surface erosion than fluoride-treated groups. Post-treatment with nano-HA results in a denser and more uniform surface layer with fewer structural defects. Similarly, enamel treated with nano-HA after cola exposure showed a statistically significant reduction in surface roughness compared with the fluoride group (*p* < 0.05). These findings suggest that nano-HA provides greater protective effects against acid-induced enamel surface erosion. Furthermore, nano-HA indicated potential antibacterial activity against *S. aureus* and *E. coli*. Overall, nano-HA toothpaste provided enhanced protection against acid-induced enamel erosion with additional antibacterial effects.

## 1. Introduction

Dental erosion is defined as the progressive loss of tooth surface resulting from acidic exposure, independent of bacterial activity. Frequent consumption of acidic beverages such as carbonated soft drinks, fruit juices, and sports drinks is a major contributing factor [1,2]. Acidic exposure initiates enamel demineralization by dissolving essential minerals, particularly calcium and phosphorus, which are key constituents of hydroxyapatite, the primary mineral component of tooth enamel. This demineralization process can lead to dentin hypersensitivity and an increased risk of dental caries [3,4].

Fluoride is widely recognized as an effective remineralizing agent with well-established caries-preventive properties. It promotes the formation of a protective fluorapatite layer, which enhances enamel resistance to acid attacks [5,6]. However, excessive mineral deposition on the surface may act as a barrier, restricting the diffusion of calcium and phosphate ions into deeper enamel layers and potentially limiting complete remineralization. Additionally, high fluoride intake during tooth development can result in dental fluorosis [7,8]. In recent years, nano-hydroxyapatite (nano-HA) has emerged as a promising biomimetic alternative. Chemically and structurally similar to natural enamel, nano-HA has demonstrated the ability to penetrate the deeper layers of enamel and fill surface defects in demineralized areas. Its nanoscale dimensions provide a high surface area and strong affinity for enamel, potentially enabling the restoration of enamel’s original microstructure [9,10]. Our previous study successfully synthesized a self-antibacterial nano-hydroxyapatite (nano-HA) via a microwave-assisted combustion method [11].

Previous studies on oral care materials, including fluoride- and nano-hydroxyapatite-based formulations, have primarily focused on individual protective aspects, particularly enamel remineralization, with comparatively limited evaluation of resistance to acid-induced erosion and antibacterial performance within a unified framework. However, effective tooth protection requires a multifunctional approach integrating remineralization, erosion resistance, and antibacterial activity.

In this study, synthesized self-antibacterial nano-hydroxyapatite (nano-HA) was incorporated into a toothpaste formulation and applied to enamel surfaces to investigate its protective effect against erosion induced by an acidic soft drink. Erosive changes in enamel surface morphology were primarily evaluated through qualitative analysis using scanning electron microscopy (SEM) and compared with those observed following treatment with a conventional fluoride toothpaste. The surface roughness was estimated using a semi-quantitative approach based on SEM image analysis using the SurfCharJ plugin in ImageJ software. Furthermore, a preliminary assessment of the antibacterial activity of the synthesized self-antibacterial nano-HA toothpaste formulation against bacterial strains was carried out.

## 2. Results and Discussion

**Figure 1:**
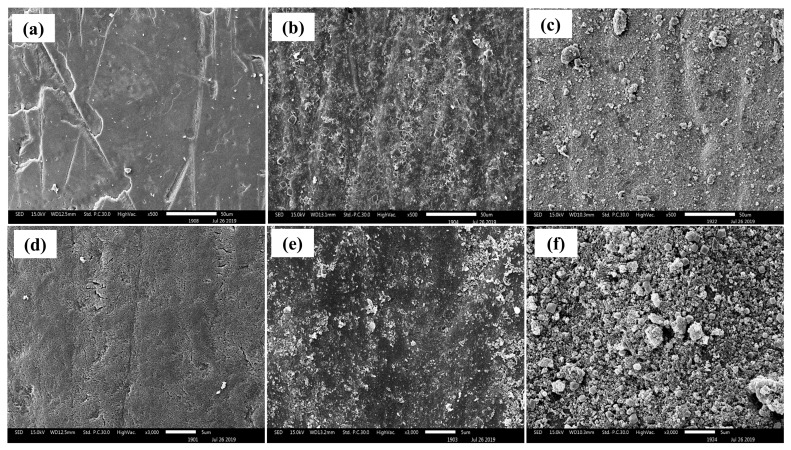
Surface morphologies of untreated enamel (**a**,**d**), enamel after pre-treatment with commercial fluoride toothpaste (**b**,**e**), and enamel after pre-treatment with synthesized nano-HA-containing toothpaste (**c**,**f**).

Scanning electron microscopy (SEM) images of the untreated enamel specimens (Group 1), taken at magnifications of 500× and 3000× (Figure 1a,d, respectively), reveal a relatively smooth and intact surface morphology. The enamel exhibits a homogeneous structure, with no visible signs of porosity, cracks, or other surface defects. Following toothpaste treatment, distinct morphological changes were observed on the enamel surfaces. In Group 2, treated with commercial fluoride toothpaste (Figure 1b,e), the enamel showed a heterogeneous surface along with scattered particulate deposits. These are likely newly formed mineral phases, such as fluorapatite, resulting from fluoride-enhanced precipitation of calcium and phosphate. In Group 3, treated with synthesized nano-hydroxyapatite (nano-HA) toothpaste (Figure 1c,f), the surface appeared more irregular, with dense granular accumulations consistent with direct deposition of hydroxyapatite particles from the toothpaste.

**Figure 2:**
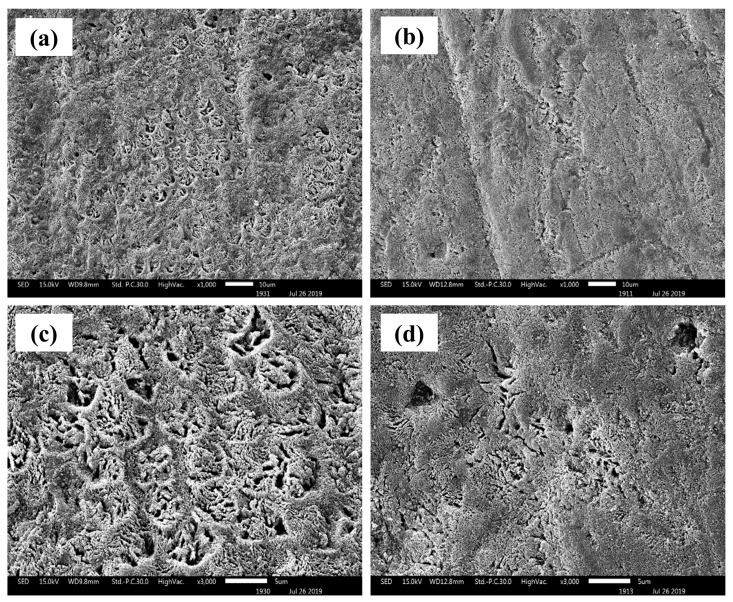
Surface morphologies of demineralized enamel pre-treated with commercial fluoride toothpaste (**a**,**c**) and with the synthesized nano-HA-containing toothpaste (**b**,**d**).

Exposure to an acidic beverage such as cola caused notable demineralization and structural damage to the enamel surface. As illustrated in Figure 2, enamel samples from Group 4 (pre-treated with fluoride toothpaste before cola exposure; Figure 2a,c) and Group 5 (pre-treated with nano-HA toothpaste before cola exposure; Figure 2b,d) exhibited surface irregularities characterized by porosity and microcracks, indicative of acid erosion. Notably, fluoride pre-treatment (Group 4) was less effective in preventing enamel erosion, as evidenced by pronounced surface damage. In contrast, nano-HA pre-treatment (Group 5) resulted in comparatively less surface deterioration, suggesting that nano-HA provided better protection against acid-induced demineralization. These findings are consistent with previous reports on dental erosion caused by acidic beverages. For instance, R.A. Jameel et al. [12] demonstrated that exposure to beverages such as cola and orange juice significantly contributes to enamel surface destruction. SEM images in their study revealed irregular enamel structures resulting from the dissolution of enamel rods and inter-rod substances. Similarly, a study conducted by N. Kumar et al. [13] reported significant changes in enamel surface morphology after immersion in various acidic beverages, including carbonated drinks, energy drinks, and fruit juices. Erosive defects such as cracks, porosity, and irregular surface patterns were observed and were attributed to the loss of mineral components in enamel specimens. Additionally, P. Sooksompien et al. [14] investigated the effects of carbonated soft drinks on primary enamel and observed severe surface damage after cola exposure, characterized by coral network-like and map-like erosive patterns.

**Figure 3:**
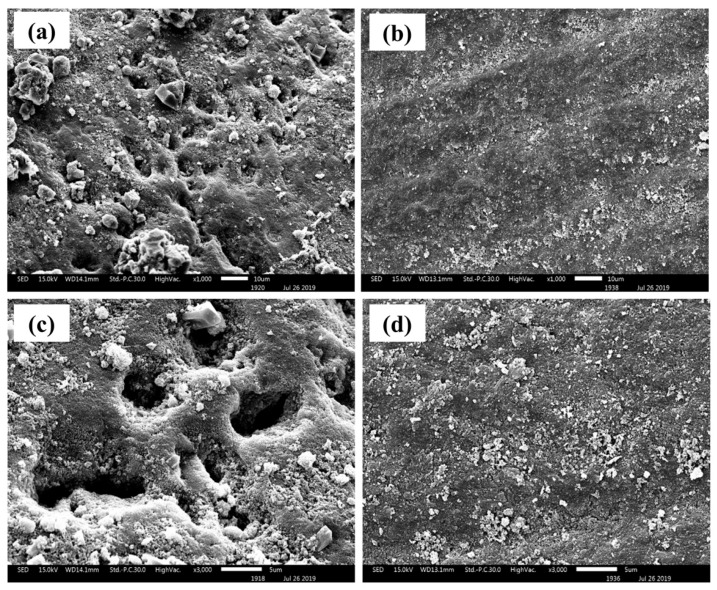
Surface morphologies of remineralized enamel after post-treatment with commercial fluoride toothpaste (**a**,**c**) and after treatment with the synthesized nano-HA-containing toothpaste (**b**,**d**).

Post-treatment with the respective toothpastes (Groups 6 and 7) further demonstrated the remineralization potential of each formulation. While both treatments contributed to enamel surface repair, the nano-HA treatment (Group 7) exhibited denser surface coverage with fewer observable defects. Fluoride-treated enamel (Figure 3a,c) exhibited only partial surface coverage, with persistent porosity and roughness, suggesting limited remineralization. This is consistent with the known mechanism of fluoride, which facilitates the conversion of hydroxyapatite to fluorapatite by attracting calcium and phosphate ions from saliva. However, this reaction typically occurs at the nanoscale and does not result in a visible surface buildup. Instead, fluorapatite forms a thin, subsurface layer that enhances acid resistance but provides minimal structural repair. In contrast, enamel treated with synthesized nano-hydroxyapatite (Figure 3b,d) showed a substantially more uniform and continuous surface. The presence of dense, granular mineral deposits indicates that nano-HA particles were directly incorporated onto the enamel surface, effectively filling in defects and creating a visibly thicker remineralized layer. This supports the idea that nano-HA acts not only as a remineralization promoter but also as a structural filler, mimicking the natural apatite crystals in enamel and contributing to more homogeneous surface coverage. These findings underscore the dual benefit of nano-hydroxyapatite (nano-HA) in both repairing and protecting demineralized enamel, offering a more robust and functionally effective restoration compared to conventional fluoride treatment. The results support the hypothesis that nano-HA facilitates more efficient enamel remineralization, particularly following acid-induced demineralization. This enhanced performance is likely attributed to the nanoscale dimensions and high surface reactivity of the synthesized HA particles, which facilitate their incorporation into enamel surface defects and promote effective mineral deposition within demineralized zones.

**Figure 4:**
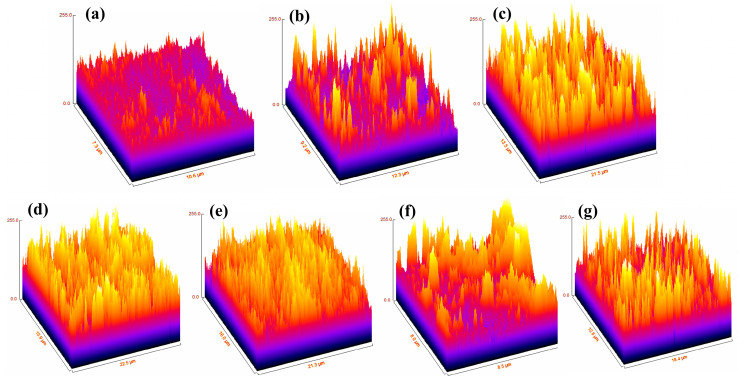
3D surface roughness plots of enamel specimens in different study groups; (**a**) Group 1, (**b**) Group 2, (**c**) Group 3, (**d**) Group 4, (**e**) Group 5, (**f**) Group 6, and (**g**) Group 7.

**Figure 5:**
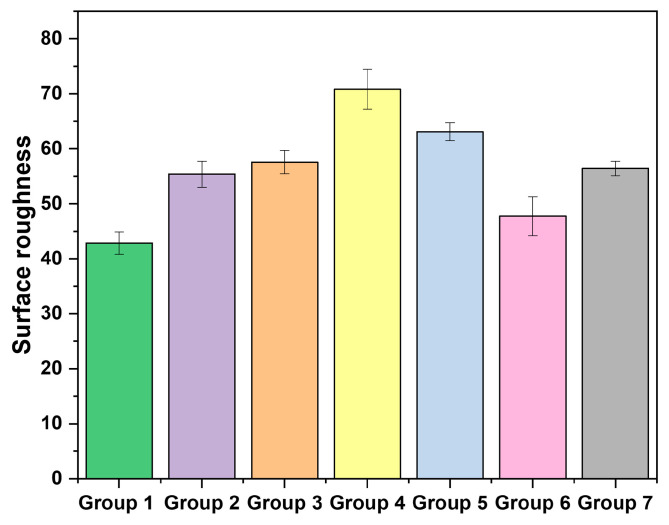
Comparison of average surface roughness values (Ra) of enamel for all study groups.

The surface roughness was estimated using a semi-quantitative approach based on SEM image analysis with ImageJ and expressed as average surface roughness (Ra), calculated with the SurfCharJ plugin. Three-dimensional (3D) surface roughness plots were generated, as shown in Figure 4. Ra values were obtained from multiple randomly selected areas within each group to ensure representative and reliable measurements. A comparison of the mean surface roughness among all groups is presented in Figure 5. One-way ANOVA analysis revealed a significant difference in surface roughness between all experimental groups and the control group (*p* < 0.05). The untreated enamel specimens (Group 1) exhibited the lowest Ra values among all groups, indicating a relatively smooth and intact surface. Following toothpaste treatment (Groups 2 and 3), an increase in surface roughness was observed, suggesting that toothpaste application increased surface irregularities, possibly due to mineral deposition on the enamel surface after treatment. In contrast, immersion in the cola beverage (Groups 4 and 5) resulted in a pronounced increase in Ra values, with these groups exhibiting the highest surface roughness overall, indicating enamel demineralization and erosion due to the acidic challenge. Notably, enamel treated with fluoride toothpaste after cola exposure (Group 4) showed a significantly greater increase in surface roughness compared with the nano-hydroxyapatite (nano-HA)-treated group (Group 5) following acidic exposure (*p* < 0.05). These findings suggest that nano-HA provides greater protective effects against acid-induced enamel surface erosion. In addition, the post-treatment groups (Groups 6 and 7) showed a significant decrease in surface roughness (*p* < 0.05) compared with their corresponding no post-treatment groups (Groups 4 and 5), indicating partial surface recovery and improved enamel smoothness after toothpaste application. These findings are consistent with the in vitro study by G. Alfinsoy and D. Celyhan [15], in which enamel surface roughness after demineralization was the highest among all groups, and a significant decrease in surface roughness was observed following toothpaste treatment. Similarly, in the study conducted by P. Li et al. [16], the surface roughness of enamel increased markedly with increasing immersion time in acidic soft drinks.

**Figure 6:**
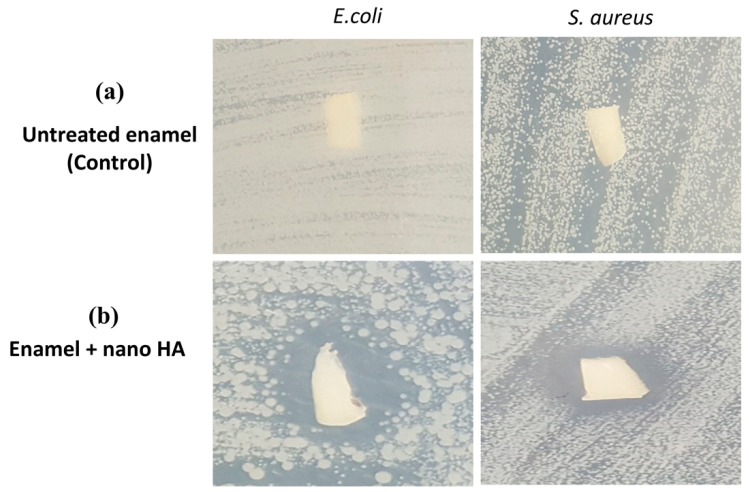
Antibacterial activity of untreated enamel (**a**), and (**b**) enamel treated with a toothpaste containing self-antibacterial synthesized nano-hydroxyapatite (nano-HA) against *S. aureus* and *E. coli*.

The antibacterial activity was evaluated by measuring the diameter of the inhibition zones. The results showed that enamel samples treated with nano-HA toothpaste exhibited distinct zones of inhibition against *S. aureus* and *E. coli* (Figure 6). The average inhibition zone diameters were 34.3 and 28.2 mm, respectively (Table 1). In contrast, no antibacterial activity was observed in the untreated enamel control group. These results indicate that the synthesized nano-HA imparts antibacterial properties to the treated enamel surface.

However, this study has several limitations that should be acknowledged. First, the relatively small sample size may limit the generalizability of the findings. Second, the evaluation was primarily based on qualitative observations of surface morphology using scanning electron microscopy (SEM), and quantitative analysis was not performed. Third, the experimental design focused on short-term erosive challenges under controlled laboratory conditions, which cannot fully replicate the complexity of the oral environment. In addition, the antibacterial activity was assessed only through qualitative in vitro observations. Therefore, future studies should include larger sample sizes and incorporate more comprehensive quantitative analyses, such as elemental composition analysis (Ca:P ratio), lesion depth measurements, surface microhardness testing, and appropriate statistical evaluations to strengthen the reliability of the results. Further studies should also include quantitative antibacterial assays, such as colony-forming unit (CFU) counting and bacterial viability assays, to provide a more objective evaluation of antibacterial efficacy against clinically relevant oral microorganisms, including streptococcus mutans and other cariogenic or periodontal pathogens. For long-term assessment, additional parameters such as simulated salivary flow conditions, brushing abrasion testing, and pH cycling models should be investigated to determine the stability and sustained effectiveness of the nano-HA formulation.

## 3. Materials and Methods

### 3.1. Preparation of Tooth Samples

The extracted human premolars (*n* = 30) used in this study were obtained under a protocol approved by the Human Experimentation Committee, Faculty of Dentistry, Chiang Mai University (Approval No. 34/2022). The protocol covered the collection and research use of extracted teeth for the evaluation of nano-hydroxyapatite-based materials. All specimens were irreversibly anonymized and could not be linked to any identifiable individual; additional ethical approval or informed consent was not required in accordance with institutional guidelines. The collected teeth were thoroughly cleaned to remove any remaining soft tissue and stored in a 10% formalin solution until use. Prior to sectioning, the teeth were rinsed with deionized water. Each tooth was carefully sectioned from buccal and lingual surfaces to obtain enamel specimens, which were then divided into two equal halves using a Dremel high-speed rotary tool. All specimens were then rinsed and ultrasonically cleaned in deionized water to remove residual contaminants on the tooth surface. Finally, the samples were air-dried at room temperature.

### 3.2. Preparation of Nano Hydroxyapatite (Nano-HA) and Formulation of Toothpaste

Self-antibacterial nano-hydroxyapatite was prepared using a microwave-assisted combustion method. The preparation procedure was based on our previous study [11]. Briefly, Ca(NO_3_)_2_ was dissolved in a H_2_O_2_ solution, followed by the addition of H_3_PO_4_. The pH of the suspension was adjusted to 10 using NH_4_OH, and the mixture was stirred for 30 min. The suspension was then heated in a microwave oven and subsequently calcined at 600 °C for 2 h. Finally, nano-hydroxyapatite (nano-HA) was obtained. The toothpaste containing hydroxyapatite (HA) was formulated by incorporating synthesized nano-hydroxyapatite (nano-HA) at a concentration of 8 wt% as the primary active ingredient. In addition to nano-HA, the formulation included conventional excipients commonly used in toothpaste, such as abrasives, humectants and flavoring agents. A commercially available fluoride toothpaste containing 1450 ppm fluoride (as sodium fluoride, NaF) was used as the reference control for comparison in this study.

### 3.3. Demineralization and Remineralization Studies

To investigate the processes of demineralization and remineralization, a cola soft drink (Coca-Cola) was used as the demineralizing agent. The demineralization process or erosive challenge protocol was adapted, with minor modifications, from the method described by M. Colombo et al. [2], which has previously been used to simulate daily soft drink consumption. The enamel specimens were immersed in the cola drink for 2 min at room temperature, followed by rinsing with deionized water. This procedure was repeated at four time intervals (0, 4, 8, 24 h). The pH of the cola drink was 2.92. For remineralization process, two types of toothpaste were employed: one containing fluoride and the other containing self-antibacterial synthesized nano-hydroxyapatite (nano-HA). The toothpaste was applied on the enamel surface at 0, 4, 8 and 24 h.

The enamel specimens were divided into seven groups based on the treatment protocol as follows. The number of enamel specimens per group was five (*n* = 5).

Group 1: Untreated (control).

Group 2: Pre-treated with a commercial fluoride-containing toothpaste for 2–3 min using an electric toothbrush, followed by rinsing with deionized water (fluoride pre-treatment). The toothpaste was applied four consecutive times.

Group 3: Pre-treated with a synthesized nano-HA-containing toothpaste under the same conditions as Group 2 (nano-HA pre-treatment).

Group 4: Enamel pre-treated with fluoride toothpaste subsequently immersed in 6 mL of cola for 2 min at room temperature, followed by rinsing with deionized water (fluoride pre-treatment + cola exposure).

Group 5: Enamel pre-treated with HA toothpaste subsequently immersed in cola under the same conditions as Group 4 (nano-HA pre-treatment + cola exposure).

Group 6: Enamel pre-treated with fluoride toothpaste, after cola drink immersion, were subsequently treated with the fluoride-containing toothpaste (demineralized enamel + post-treatment with fluoride toothpaste).

Group 7: Enamel pre-treated with HA toothpaste, after cola drink immersion, were subsequently treated with the nano-HA-containing toothpaste (demineralized enamel + post-treatment with nano-HA toothpaste).

### 3.4. Characterization of Tooth Samples

A scanning electron microscope (SEM, JEOL JSM-IT300, JEOL Ltd., Tokyo, Japan) was used to observe the qualitative changes in the surface morphology of the enamel specimens in each experimental group. The specimens were mounted on stubs using carbon tape and coated with gold using a sputter coater. SEM images of the enamel surfaces at each experimental group were taken at different magnifications (500×, 1000× and 3000×). Surface roughness was estimated using a semi-quantitative approach based on SEM image analysis with ImageJ software version 1.54r and expressed as average roughness (R_a_), calculated with the SurfCharJ plugin version 1q, as reported in several previous studies [17,18,19]. R_a_ values were obtained from multiple randomly selected areas for each group.

### 3.5. Preliminary Study for Antibacterial Activity

The antibacterial activity of enamel treated with a toothpaste containing self-antibacterial synthesized nano-hydroxyapatite (nano-HA) against Gram-positive bacteria (*S. aureus*) and Gram-negative bacteria (*E. coli*) was evaluated using the agar diffusion assay. In the experiment, the bacterial suspension was adjusted to the appropriate concentration by matching its turbidity to McFarland standard No. 0.5. The bacteria were then spread onto nutrient agar plates, and the enamel specimens were placed on the surface of the agar. The plates were incubated for 24 h, after which the diameter of the inhibition zone (or clear zone) was measured. The presence of a clear zone indicated inhibition of bacterial growth. A control experiment using untreated enamel was also conducted under the same conditions for comparison. The experiment was performed in triplicate to ensure reproducibility, and the relative standard deviation was less than 5%. The data are presented as the average inhibition zone diameter.

### 3.6. Statistical Analysis

Statistical analysis of the experimental data was performed using Microsoft Excel with the Data Analysis add-in. One-way analysis of variance (ANOVA) was used to determine whether statistically significant differences in surface roughness existed among the studied groups. When significant differences were detected, pairwise comparisons between specific groups were conducted using a post hoc test. A significance level of *p* < 0.05 was considered statistically significant.

## 4. Conclusions

The findings of this study, based on qualitative SEM surface morphology observations, suggest a potential protective effect of a toothpaste formulation containing synthesized self-antibacterial nano-hydroxyapatite (nano-HA) against enamel surface erosion induced by a cola-based soft drink. Enamel specimens treated with nano-HA toothpaste after cola exposure showed more continuous surface coverage and fewer visible erosive features under SEM evaluation. Surface roughness analysis further revealed that these specimens showed a statistically significant reduction in roughness values compared with the fluoride group (*p* < 0.05), indicating a greater resistance to acid-induced surface erosion. In addition, nano-HA-treated enamel showed antibacterial activity against *S. aureus* and *E. coli*. Overall, these results suggest that nano-HA-containing toothpaste may offer potential benefits in enamel surface protection and antibacterial performance. The findings support further investigation of nano-HA-based formulations as a promising next-generation strategy for comprehensive enamel protection. However, as the present conclusions are derived from qualitative in vitro observations, additional quantitative, well-controlled, and long-term studies are necessary to confirm these findings and establish their clinical relevance.

## Figures and Tables

**Table 1:** Inhibition zone diameters (mm) of enamel treated with a toothpaste containing self-antibacterial synthesized nano-hydroxyapatite (nano-HA) against bacterial strains.

	*E. coli*	*S. aureus*
Control (untreated enamel)	0.00	0.00
Enamel treated with toothpaste containing nano-HA	34.3	28.2

## Data Availability

The original contributions presented in this study are included in the article. Further inquiries can be directed to the corresponding author.
